# Treatment and outcomes of chronic locked posterior shoulder dislocations: a retrospective case series

**DOI:** 10.1186/s12891-023-06200-8

**Published:** 2023-01-31

**Authors:** Emil George Haritinian, Ioan Cristian Stoica, Roman Popescu, Gavril Lucian Gheorghievici, Laurent Nové-Josserand

**Affiliations:** 1 Carol Davila University of Medicine and Pharmacy, 37 Dionisie Lupu, 020021 Bucharest, Romania; 2 Foișor Orthopaedic Hospital, 35-37 Ferdinand I, 021382 Bucharest, Romania; 3 Ramsay Santé, Hôpital Privé Jean Mermoz, Centre Orthopédique Santy, 24 Avenue Paul Santy, 69008 Lyon, France

**Keywords:** Posterior shoulder dislocation, Locked shoulder dislocation, Chronic shoulder dislocation, Modified Mclaughlin

## Abstract

**Background:**

Posterior shoulder dislocations are rare injuries that are often missed on initial presentation. Cases left untreated for more than three weeks are considered chronic, cannot be reduced closely (they become locked) and are usually associated with a significant reverse Hill-Sachs defect. The aim of this study was to evaluate the outcomes of chronic locked posterior shoulder dislocations treated with the McLaughlin procedure (classic or modified).

**Methods:**

This retrospective study included 12 patients with chronic locked posterior shoulder dislocation operated on between 2000 and 2021 by two surgeons in two institutions. Patients received a thorough clinical examination and radiological assessment before and after surgery. Shoulders were repaired with the McLaughlin or modified McLaughlin procedure. Outcomes were assessed by comparing pre- and postoperative values of clinical variables.

**Results:**

Most of the dislocations were of traumatic origin. The average delay between dislocation and surgical reduction was 13.5 ± 9.7 weeks. Postoperative clinical outcomes were favourable, with an average subjective shoulder value of 86.4 ± 11.1 and a normalized Constant –Murley score of 90 ± 8.3. None of the patients had a recurrence of shoulder dislocation, but one patient developed avascular necrosis of the humeral head and two patients developed glenohumeral osteoarthritis.

**Conclusions:**

In this group of patients with chronic locked posterior shoulder dislocation, the clinical outcomes of McLaughlin and modified McLaughlin procedures were satisfactory, even when surgery was significantly delayed.

## Background

Posterior shoulder dislocation is a rare injury, accounting for just 2 to 5% of all shoulder dislocations [1–3]. The most common causes are convulsions (epileptic, hypoglycaemic or drug withdrawal), trauma and electric shocks [3, 4]. A significant percentage (50–79%) of posterior shoulder dislocations are missed on initial presentation [5]. Thorough clinical examination is crucial and usually shows flattening of the anterior shoulder, a prominent coracoid process, severely limited external rotation and reduced abduction [6]. True anteroposterior and axillary or scapular Y radiographic views of the affected shoulder are also required. Several diagnostic signs (light bulb, vacant glenoid, double trough, rim) have been described on anteroposterior radiographs [5, 7].

Posterior shoulder dislocations are associated in 30 to 90% of cases [8] with an osteochondral impression fracture of the anteromedial part of the humeral head called a reverse Hill-Sachs lesion (RHSL) [9]; however, the term posterior shoulder *fracture-*dislocation is usually only used in cases (about one third [10]) with fractures of the humeral neck or the tuberosities [11]. Alternatively, Robinson et al. [12] have suggested referring to the latter as "complex" fracture-dislocations, as opposed to "simple" fracture-dislocations involving only an impression fracture (RHSL).

Computed tomography (CT) is required to evaluate the RHSL and other associated fractures and for preoperative planning. Irreducible dislocations with an RHSL are called "locked" or "fixed" [5], and cases neglected for more than three weeks are considered "chronic" [4]. Reverse Hill-Sachs lesions continue to grow while the shoulder remains dislocated [13].

The choice of procedure and prognosis depend on the time to surgery, the size of the RHSL and the possible presence of glenohumeral osteoarthritis. Conservative treatment can be effective in promptly diagnosed cases with stable shoulders and minor bone defects (less than 25% of the articular surface) after closed reduction [6]. Locked posterior shoulder dislocations with an RHSL involving 25–40% of the articular surface can be repaired by transposition of the subscapularis muscle (McLaughlin procedure) [14, 15], lesser tuberosity transposition (Hawkins et al.’s modified McLaughlin procedure) [15–17], a modified McLaughlin procedure augmented with an autograft from the iliac crest [18], reconstruction of the humeral head defect using an allograft [19, 20], rotational osteotomy of the humerus [21, 22] or posterior bone block [23]. Cases in which the RHSL involves more than 40% of the articular surface are usually treated by shoulder replacement surgery, either hemiarthroplasty [24], anatomic total shoulder arthroplasty [24, 25], or reverse total shoulder arthroplasty [26].

The purpose of this study was to evaluate the outcomes of open reduction and stabilisation of chronic locked posterior shoulder dislocations using the McLaughlin or modified McLaughlin procedure.

## Methods

### Study design

This was a retrospective investigation of twelve patients with chronic locked posterior shoulder dislocations operated on between 2000 and 2021 by two surgeons (LNJ and EGH) in two institutions (Hôpital Privé Jean Mermoz, Lyon, France and Foișor Orthopaedic Hospital, Bucharest, Romania).

### Pre- and postoperative data

**Fig. 1:**
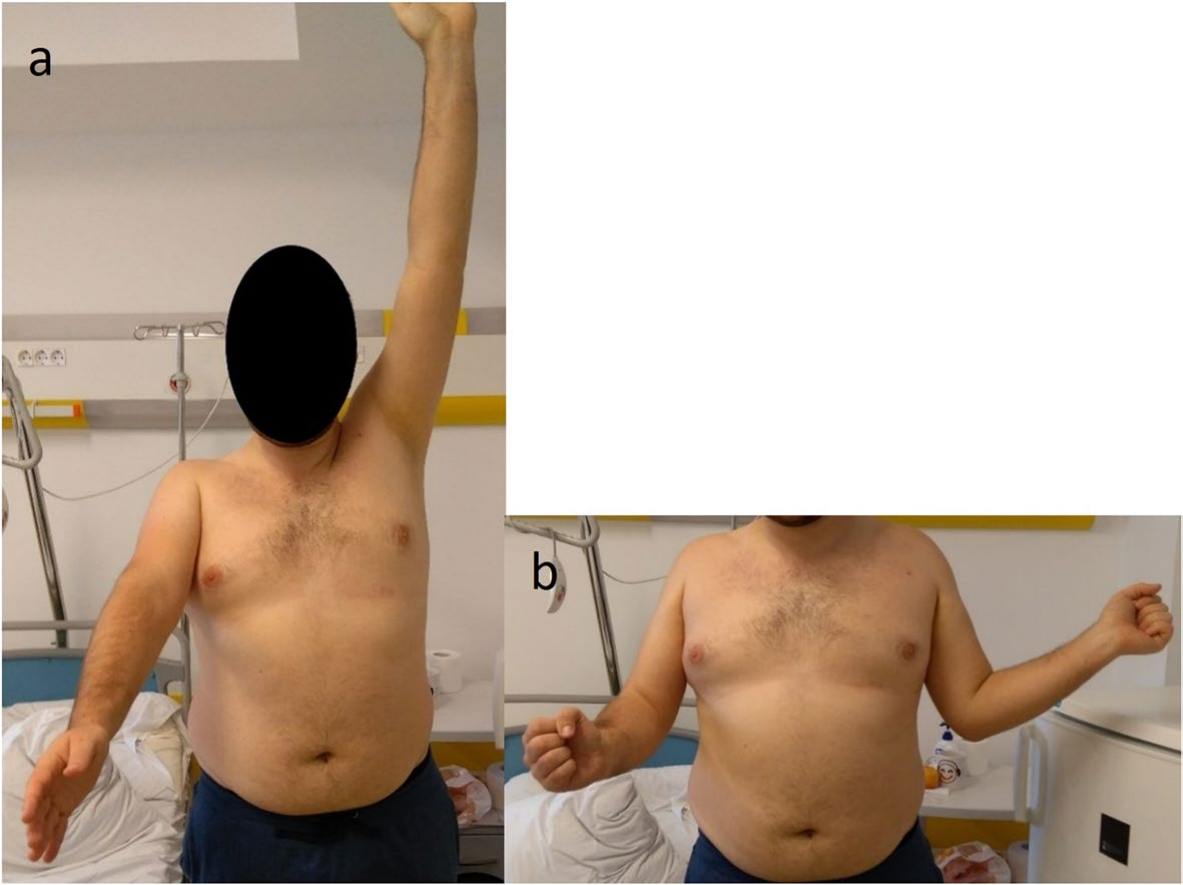
Locked posterior shoulder dislocation (right shoulder), preoperative physical examination. Photographs of the right arm of a patient in (**a**) active anterior elevation and (**b**) active external rotation elbow at side

**Fig. 2:**
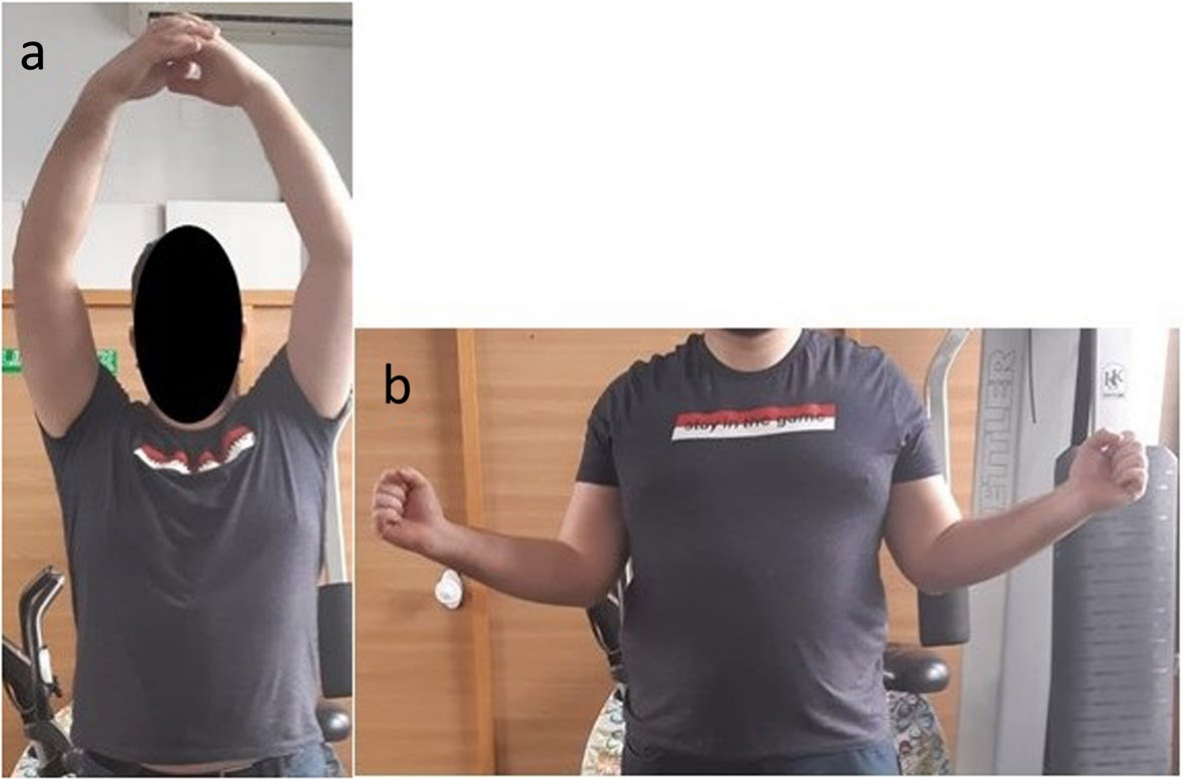
Locked posterior shoulder dislocation (right shoulder), postoperative physical examination. Photographs of the right arm of a patient with locked posterior shoulder dislocation treated with the modified McLaughlin procedure in (**a**) active anterior elevation and (**b**) active external rotation elbow at side

**Fig. 3:**
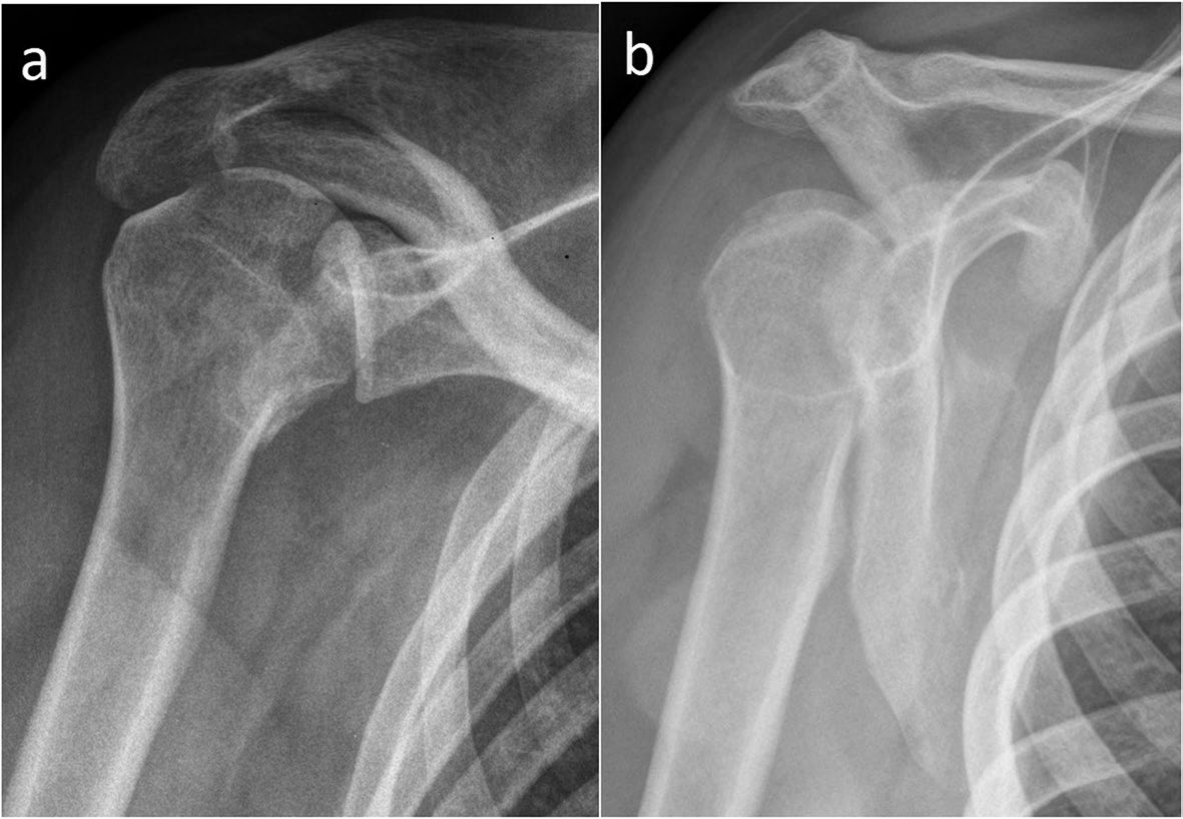
Locked posterior shoulder dislocation (right shoulder), preoperative imaging. **a** Anteroposterior and (**b**) scapular Y view X-ray images of the right shoulder of a patient with locked posterior shoulder dislocation

**Fig. 4:**
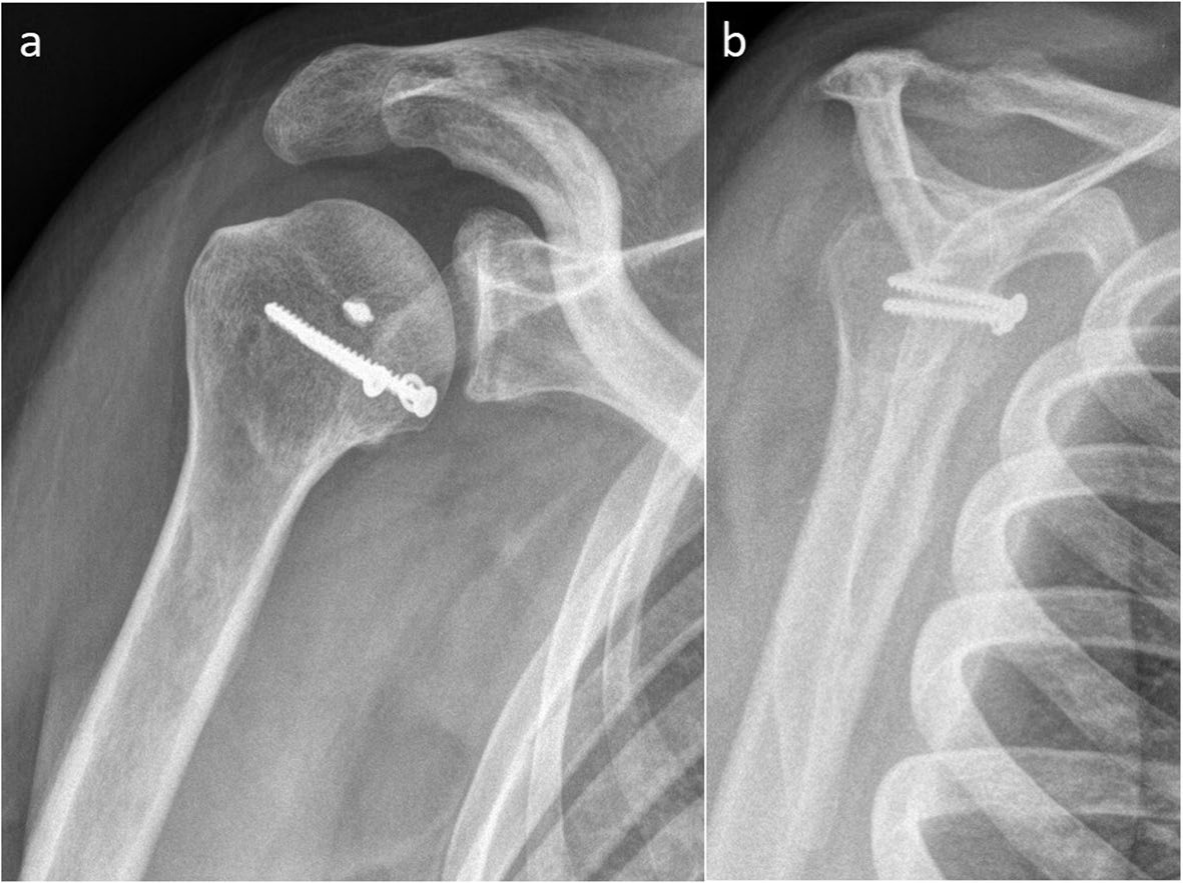
Locked posterior shoulder dislocation (right shoulder), postoperative imaging. **a** Anteroposterior and (**b**) scapular Y view X-ray images of the right shoulder of a patient with locked posterior shoulder dislocation treated with the modified McLaughlin procedure

**Fig. 5:**
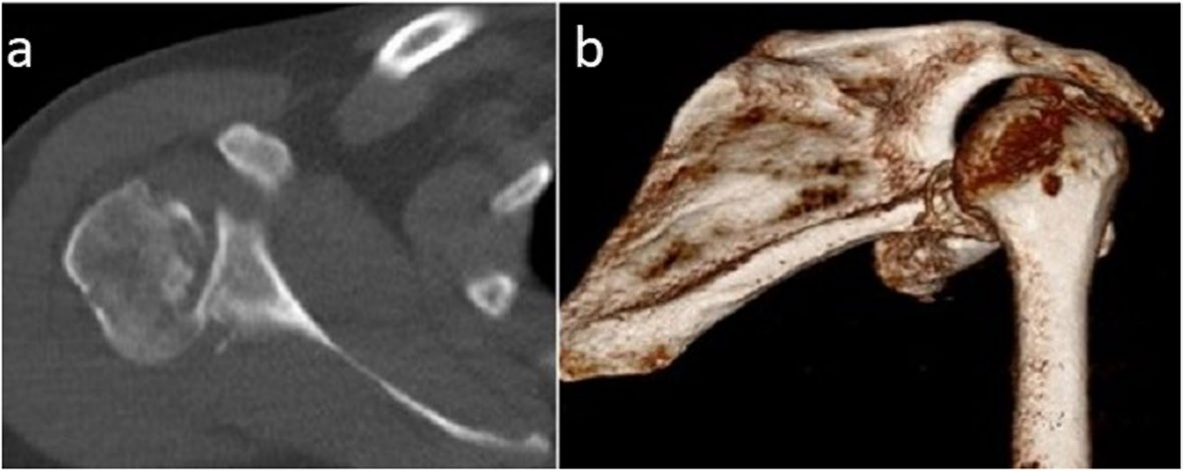
Locked posterior shoulder dislocation (right shoulder), preoperative imaging. **a** Axial and (**b**) three-dimensional reconstructed computed tomography images of the right shoulder of a patient with locked posterior shoulder dislocation

**Fig. 6:**
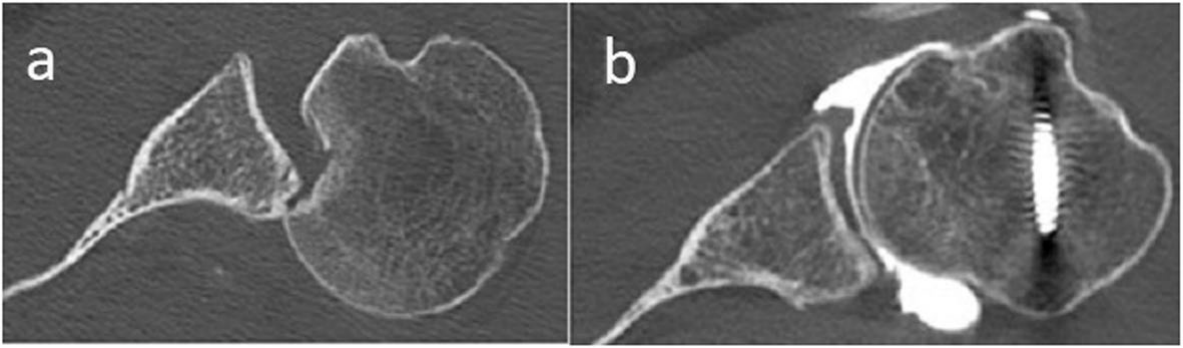
Locked posterior shoulder dislocation (left shoulder), pre- versus postoperative imaging. **a** Preoperative and (**b**) postoperative axial computed tomography images of a the left shoulder of a patient with locked posterior shoulder dislocation treated with the modified McLaughlin procedure

The data considered included the mechanism of injury, the active range of motion of the shoulder before and after surgery (anterior elevation, abduction, external rotation elbow at side, and internal rotation (Figs. 1 and 2)) pre- and postoperative Constant–Murley scores [27, 28], and subjective results at last follow-up (satisfaction on a 5-point Likert scale and subjective shoulder value (SSV) scores [29]). Internal rotation was evaluated using the Constant–Murley scale [27, 28] based on the highest position reached by the patient’s thumb on their back. Strength in external rotation was measured using the Medical Research Council Manual Muscle Testing scale [30]. Shoulder X-rays (anteroposterior and scapular Y images*,* Figs. 3 and 4) and CT scans (Figs. 5 and 6) were also evaluated. We measured the size of the defect on CT-scans in the axial plane (alpha axial angle) using the method described by Moroder et al. [31]. We estimated the percentage of the involved articular surface, in the axial plane, considering the angular defect on the humeral joint surface in relation with the entire humeral articular surface [31]. Post-operatively, all patients were followed-up radiologically (x-rays and in two cases CT-scans), however we did not evaluate the healing of the subscapularis using ultrasonography or MRI.

### Surgery

All operations were performed using a deltopectoral approach, with the patient under general anaesthesia in the beach chair position.

In patients treated with the McLaughlin procedure (*n* = 2), the cranial two-thirds of the subscapularis tendon was released from its insertion on the lesser tuberosity. The tendon was marked with strong non-absorbable suture and was retracted medially. The RHSL and the glenoid were inspected after removing all fibrous tissue present. A blunt Hohmann retractor was placed between the humeral head and the glenoid. The joint was reduced using lateral distraction and gradual external rotation and the remaining humeral head cartilage and RHSL were evaluated. The base of the RHSL was rasped to obtain a bleeding surface. The subscapularis tendon was then transferred inside the RHSL, as close as possible to the medial edge, using either transosseous sutures (one case) or suture anchors (one case).

In patients treated with the modified McLaughlin procedure (*n *= 10), the lesser tuberosity was osteotomised laterally from the bicipital groove up to the level of the RHSL. The subscapularis tendon was tagged with strong non-absorbable suture and the lesser tuberosity was retracted medially. After inspecting the joint and RHSL and preparing the RHSL as described above, the lesser tuberosity was transferred inside the RHSL, close to its medial edge and fixed with cancellous screws and washers. An additional anchor was inserted in the RHSL in four cases (metallic in two cases, resorbable in two cases) along with sutures passed through the subscapularis tendon. No graft was used.

The stability of the joint was evaluated at the end of the procedure.

### Postoperative management

Patients wore a shoulder brace maintaining the shoulder in neutral or external rotation (mainly depending on the available brace type) for 4 to 6 weeks. Hand and elbow mobilisation began from the first day after surgery. Shoulder rehabilitation was started after removal of the brace, beginning with self-rehabilitation and passive mobilisation. Active shoulder mobilization was started at about 6 weeks for patients immobilized for 4 weeks and at about 8 weeks for patients immobilized for 6 weeks.

### Statistical analysis

Categorical variables were summarised as frequencies. Continuous variables were expressed as mean ± standard deviation and compared before and after surgery using paired t tests. Independent samples t tests were run to compare the postoperative results. Differences were considered statistically significant at *p* < 0.05. All analyses were performed using IBM SPSS Statistics, version 24.

## Results

The average age at surgery was 48.2 ± 13.3 years (range, 30–67 years). Ten of the twelve patients were men. The average delay between injury and surgery was 13.5 ± 9.7 weeks (range, 3–32 weeks).

The cause of the dislocation was in one case, electrocution, in one case reflex epilepsy, in one case, unclear (apparently, the dislocation occurred when the patient rolled over in bed), and in the nine remaining cases, trauma (three motorcycle accidents, and six falls).

The initial dislocation was associated with a humeral fracture (other than the RHSL) in 8 of the 12 patients (66.7%), an isolated lesser tuberosity fracture in three cases, an oblique fracture of the proximal third of the humerus in two cases, and in one case each a spiral humeral shaft fracture, a fracture of the surgical neck and lesser tuberosity, and a multifragmentary fracture of the proximal humerus including the surgical neck, and the lesser and greater tuberosity. In all these cases of complex posterior shoulder fracture-dislocations, the associated fractures were initially treated conservatively. The posterior dislocation was missed on initial presentation in all but one case, in which, after closed reduction, the dislocation recurred. The associated fractures had healed at the time of surgery in six of eight cases because the patients presented late. In the two remaining cases, the unhealed lesser tuberosity fragment was fixed at the level of the RHSL.

The average alpha angle was 46.9° ± 7.9° (32.1°—59.5°). The RHSL represented on average 25.6% ± 5% (17.1%-36%) of the articular surface.

The McLaughlin procedure was used in two cases (16.7%) and the modified McLaughlin procedure in ten cases (83.3%).

The postoperative immobilisation period was four weeks in three patients (25%) and six weeks in nine patients (75%). The shoulder was immobilised in external rotation in six patients (50%) and in neutral rotation in six patients (50%).

One of the patients, a 44-year-old male, treated with the modified Mclaughlin procedure in 2014, had the last follow-up at 43 days postoperatively. The patient was satisfied with the result, had no pain, no early complications, and the reduction was maintained. Because of the very short follow-up, we decided to exclude this patient from our clinical results. The following analysis was performed on the remaining eleven patients.

The average delay between injury and surgery was 14.2 ± 9.9 weeks (range, 3–32 weeks).

The average postoperative follow-up, when the SSV, Constant score and shoulder range-of motion were recorded, was 37 ± 58 months (range, 6–206 months).

Pre- and postoperative clinical variables are compared in Table 1*.*

**Table 1:** Clinical variables

	Before surgery	After surgery	p	Postoperative gain
Active anterior elevation	51 ± 27	142 ± 36	0.007	82 ± 55
Active abduction	21 ± 9	129 ± 31	< 0.001	105 ± 22
Active external rotation elbow at the side	-29 ± 22	38 ± 20	< 0.001	66 ± 17
Active internal rotation^a^	0.9 ± 1.1	6.0 ± 1.6	< 0.001	4.3 ± 1.4
Pain subscore^b^	5.8 ± 3.8	12.3 ± 2.6	0.017	7.5 ± 5.2
Daily activities subscore	2.8 ± 0.8	17.8 ± 2.5	0.001	15.4 ± 1.8
Mobility subscore	2.0 ± 2.0	28.9 ± 7.5	< 0.001	27.2 ± 5.9
Strength subscore	0	18 ± 5.0	< 0.001	17.3 ± 5.2
Constant score	9.8 ± 3.6	78.3 ± 10.8	< 0.001	69 ± 8.5
Normalized Constant score	10.8 ± 3.6	90 ± 8.3	< 0.001	76.2 ± 7.1

Data are reported as mean ± standard deviation

^a^the number of points for the maximal vertebral level reached by the patient’s thumb (Constant–Murley score)

^b^ evaluated using the Constant-Murley score methodology, a higher pain subscore means less pain

Six patients (54.5%) were satisfied and five patients (45.5%) were very satisfied with postoperative outcomes.

The average postoperative SSV was 86.4 ± 11.1 (range 70–100). The strength in external rotation elbow at side was grade 5 in all seven cases in which it was evaluated.

All clinical variables increased significantly between pre- and postoperative assessments (Table 1). None of the patients had a recurrence of the dislocation on the operated shoulder. There were no implant-related, infectious or neurological complications. At last follow-up (12 months), one patient had avascular necrosis of the humeral head (Cruess stage IV [32]) and two patients had glenohumeral osteoarthritis (Samilson-Prieto Allain stages 2 and 3 [33, 34], respectively).

There were no significant differences between the outcomes of patients treated with McLaughlin and modified McLaughlin procedures (independent samples t test, *p* > 0.05 for all the followed clinical variables). Also, the results of cases operated by LNJ or EGH were similar for all the observed clinical variables (independent samples t test, *p* > 0.05), with the exception of the normalized Constant score, which was better in the group of patients operated by LNJ (independent samples t test, *p* = 0.04).

## Discussion

The clinical results obtained with the original or modified McLaughlin procedures in patients with locked posterior shoulder dislocation were favourable and comparable with those of other published studies [7, 17, 18, 35].

McLaughlin called posterior shoulder dislocation a "diagnostic trap" [36] because of the high percentage of cases missed on initial presentation. This was also the case in our study, with a significant delay between dislocation and reduction (14 weeks on average). However, even with this treatment delay in a traumatic intra-articular pathology, the clinical outcomes were still favourable, with significant improvements in all clinical variables. Also, all operated shoulders remained stable. It has to be noted that in our study the RHSL was moderate on average and this might explain our good results. A larger average RHSL would have been associated with a poorer result and, in some cases, the necessity of shoulder replacement.

These outcomes contrast with the results of the wide range of open reduction and stabilisation techniques used in chronic anterior shoulder dislocations, where a high incidence of re-subluxation and early osteoarthritis have been reported [37]. The Latarjet procedure, otherwise very successful, is considered by Walch et al. [38] to be contraindicated for locked anterior shoulder dislocations. Li et al. [39] evaluated the effectiveness of the Latarjet procedure in patients with chronic anterior dislocation and found an overall rate of postoperative redislocation or subluxation of 48%. The authors noted that in cases where the reduction was possible through a subscapularis-splitting approach, no postoperative instability was encountered [39]. However, in these chronic cases it can be very difficult to obtain reduction without a subscapularis tenotomy [37, 39].

Nevertheless, missed diagnoses of posterior shoulder dislocations – and thereby in most cases, the need for surgery – could easily be avoided by increasing orthopaedic surgeons’ and emergency doctors’ awareness of this pathology.

The proportion of cases with an associated fracture (complex posterior fracture-dislocations, 73%) was comparable to the value reported in a literature review of locked posterior shoulder dislocations (75.8%) [4]. The incidence of glenohumeral osteoarthritis was higher than reported in the same review (18.2% vs. 6.7% [4]). However, one of the two patients had pre-existing osteoarthritis (Samilson-Prieto Allain stage 1 before surgery, which progressed to stage 2 at 19 months’ follow-up) and the other developed osteoarthritis (Samilson-Prieto Allain stage 3) a very long time (more than 17 years) after surgery. The latter patient was very satisfied with the result of surgery at last follow-up, and had 110° of active anterior elevation, 25° of active external rotation, elbow at the side, and could reach L3 with his thumb on internal rotation. His normalised Constant score was 98.5%. The other patient with shoulder osteoarthritis (Samilson-Prieto 2) was satisfied with the result of surgery, had 160° of active anterior elevation, 40° of active external rotation, elbow at side, could reach L5 with his thumb, and had a normalised Constant score of 81.1%. None of the patients required shoulder arthroplasty.

There was one case of avascular necrosis (9%), in keeping with the reported incidence in the literature (3.5%) [4] given the number of patients in the study. This case (Cruess stage IV) developed soon after surgery, after a long delay between dislocation and surgical reduction (23 weeks). The patient had a good clinical outcome (no pain and acceptable function) and did not require shoulder arthroplasty.

There were no postoperative subluxations or re-dislocations. The patient with epilepsy had posterior dislocation of the contralateral shoulder caused by another seizure, but the operated shoulder remained stable.

The modified McLaughlin procedure addresses the problem of poor healing of the transferred subscapularis at the level of the RHSL in the classic McLaughlin procedure. The transfer of the lesser tuberosity together with the subscapularis should lead to faster and more predictable bone-to-bone healing.

Hawkins et al. [15] achieved equally good results with McLaughlin and modified McLaughlin procedures. These authors also reported the results of five revisions of failed McLaughlin procedures performed by other surgeons and ascribed these failures either to the RHSL being too large for this technique or to the cartilage being too severely damaged at the time of the initial surgery.

The most commonly described treatments for chronic locked posterior shoulder dislocation are the McLaughlin procedure (classic or modified) and segmental reconstruction of the humeral head with a bone graft. Both are valid alternatives and have been shown to have consistently good long term outcomes and low complication rates [19, 20, 37, 40].

In our study, the classic McLaughlin procedure was used in only two cases, with a RHSL of 17.1% and 17.9%, respectively. The reduction delay was in one case 3 weeks. However, in the other case the reduction delay was 25 weeks. The clinical results in both cases were good, with a normalized Constant score of 85% and 95%, respectively. The clinical result was better in the case of the patient with a much longer reduction delay.

Proximal humerus derotational osteotomy [21, 22, 41] can also lead to good outcomes in patients without severe articular cartilage damage. However, because of the technical complexity of the procedure and the risk of humeral head necrosis and osteoarthritis progression, some surgeons only consider derotational osteotomy as a salvage procedure when there are no other treatment options [7].

Posterior bone block has also been used to treat chronic locked posterior dislocations, with satisfactory mid-term clinical results but a high rate of postoperative osteoarthritis [23].

Results could still potentially be improved by using less invasive techniques. Arthroscopic McLaughlin procedures have recently been described [42, 43]. A recent comparison [44] of the outcomes of open modified McLaughlin and arthroscopic McLaughlin procedures found that while there were no significant differences in terms of clinical results or subscapularis strength recovery, the arthroscopic McLaughlin procedure was associated with a better sense of stability and well-being.

The limitations of this study include its retrospective nature, small number of patients (which reflects the rarity of this pathology), the fact that it was conducted in only two centers and, in some cases, relatively short follow-up. As a result, this study suffers from inadequate case representation and case selective bias. However, this is a very rare pathology so most of the series in the literature have the same weaknesses.

## Conclusions

This group of patients with chronic locked posterior shoulder dislocation treated using the McLaughlin or the modified McLaughlin procedure had good clinical outcomes, even when surgical reduction was significantly delayed.

## Data Availability

The datasets used and/or analysed in this study are available from the corresponding author on reasonable request.

## Abbreviation

RHSL: Reverse Hill–Sachs Lesion
